# Mentorship as metadata: clinical annotation and the limits of real-world data in oculoplastic surgery

**DOI:** 10.3389/fopht.2026.1972892

**Published:** 2026-09-24

**Authors:** Itay Nitzan, Tehila Shlomov, Zvi Gur

**Affiliations:** 1 Department of Ophthalmology, Hadassah–Hebrew University Medical Center, Jerusalem, Israel; 2 Faculty of Medicine, Hebrew University of Jerusalem, Jerusalem, Israel

**Keywords:** causal inference, electronic health records, estimand, mentorship, oculoplastics, phenotyping, real-world data, research methodology

## Abstract

In oculoplastic surgery, real-world databases capture diagnosis and procedure codes but rarely the clinical variables that define surgical meaning: laterality, levator function, margin-reflex distance, inflammatory activity, operative intent, or the reason for revision. We propose that clinical supervision be recorded as *documented clinical interpretive annotation*: an explicit, written layer that defines the limits of routinely collected data before they are analyzed. “Mentorship as metadata” names the educational relationship that produces this annotation and is used throughout as a labelled metaphor, not as a claim of equivalence with formal metadata, data provenance, or research-governance documentation. Synthesizing established reporting and causal-inference frameworks with recently published oculoplastic database studies (2019–2026), we develop a three-layer model: code, phenotype, and judgment, together with a pre-analysis phenotype map that trainees and mentors can complete before a query is run. Central to this framework is the “refusal point”: a documented, pre-specified boundary at which a dataset should not be used to answer a given clinical question, co-signed by the oculoplastic mentor, the trainee or lead analyst, and a methodologist and recorded as a routine study-design deliverable. The refusal point complements the estimand and target-trial frameworks by naming the adjacent, more tempting question that the data cannot answer. It introduces no new statistical construct; its contribution is documentary, procedural, and educational, and we state explicitly what each existing framework already requires and what remains unspecified. We advance one falsifiable hypothesis: that pre-specified, co-signed refusal-point documentation increases pre-analysis reframing of research questions and improves concordance between the protocol estimand and the estimand the published conclusions can support, and we set out a prospective programme capable of refuting it. Real-world data remain valuable for utilization, trends, and rare associations, but they are not self-interpreting. Training oculoplastic surgeon-scientists to name, in writing and before analysis, the boundary between code and clinical reality may make database research more transparent, restrained, and reproducible. In oculoplastics, mentorship is the clinical annotation that turns data into evidence.

## Introduction

Oculoplastic surgery is a small anatomical field with a broad clinical range, spanning eyelid malposition, lacrimal disease, orbital inflammation, thyroid eye disease, trauma, tumors, reconstruction, facial nerve palsy, and aesthetic surgery. Training has therefore depended on apprenticeship: the trainee learns not only where to place an incision but also how to read asymmetry, tissue quality, scarring, expectations, function, and risk.

The subspecialty is also unusually exposed to the limits of routinely collected data when compared with other ophthalmic surgical fields. In cataract and vitreoretinal surgery, the principal endpoints (visual acuity, intraocular pressure, central subfield thickness) are quantitative, near-universally recorded, and increasingly captured in structured electronic fields and in registries designed around them. In oculoplastic surgery, the corresponding endpoints (margin-reflex distance, levator function, proptosis in millimeters, Clinical Activity Score, contour, symmetry, and patient satisfaction) are recorded in free text, in clinical photographs, or not at all, and the outcome most often analyzed, reoperation, is a utilization code rather than a clinical measurement. The gap between what is coded and what is clinically decisive is therefore wider here than in subspecialties whose endpoints were already numeric.

Ophthalmology is increasingly shaped by real-world data, defined by the U.S. Food and Drug Administration as data relating to patient health status or health-care delivery routinely collected from sources such as electronic health records, claims, registries, and digital health technologies (1). These resources offer scale, speed, longitudinal follow-up, and rare-outcome ascertainment, and are attractive in oculoplastics because many questions involve uncommon diseases, delayed revision, changing technology, or low-frequency complications. The problem is not their use, but the assumption that routinely collected data are a complete clinical reality. In oculoplastics, the variables that define diagnosis, indication, technical success, revision, and patient satisfaction are often not coded. This makes mentorship more important, not less: the mentor teaches what the dataset cannot encode.

We propose a practical educational framework for residents, fellows, early investigators, and supervising surgeons. We define the contribution narrowly, because the problem is not the existence of imperfect codes, but the absence of explicit clinical boundaries around their use. Codes are already recognized as imperfect surrogates for clinical constructs, and modern practice already demands rigor: the estimand framework of ICH E9(R1) requires investigators to define the precise effect a study targets (2), and target trial emulation requires them to pre-specify the hypothetical randomized trial that observational data approximate (3, 4).

We state at the outset what the refusal point is not. It is not a new statistical construct, and it does not identify a failure mode that existing methods cannot already name: a non-estimable estimand, a positivity violation, an unverifiable exchangeability assumption, and a construct the data do not measure are all established concepts. What is lacking is a record of that assessment. Assessment of data fitness for purpose is at present usually informal; it is performed at a variable point in the workflow, by an unspecified person, and is reported, if at all, as a limitation written after the results exist. We therefore define the *refusal point* as the pre-specified, written statement of the version of the research question that this dataset should not be used to answer, produced before data extraction, co-signed by the clinical expert, the trainee or lead analyst, and a methodologist, filed with the study protocol, and auditable against the final manuscript. Its contribution is documentary, procedural, and educational rather than statistical. We specify, for each framework we build on, what it already requires, what it leaves unspecified, and what the refusal point adds.

Our contribution is threefold: first, an oculoplastics-specific articulation of variables that are decisively uncoded in this subspecialty; second, the “refusal point” as a documented routine deliverable, defined as the pre-specified boundary at which a dataset should not be used to answer a clinical question; and third, an explicit specification of who documents that boundary, when, where it is stored, and how compliance with it can be audited. This boundary should be written before analysis and should remain a shared clinical and methodological responsibility, with the oculoplastic mentor accountable for its clinical content but not its sole owner. Throughout, we use *documented clinical interpretive annotation* for the artifact and “mentorship as metadata” as an explicitly labelled metaphor for the relationship that produces it; the two are not interchangeable, and no part of the framework depends on the metaphor.

We advance a single falsifiable hypothesis: that pre-specified, co-signed refusal-point documentation changes research behaviour and research output. We predict that oculoplastic real-world data projects completing a co-signed refusal point before data extraction will show (i) a higher frequency of pre-analysis reframing of the research question; (ii) greater concordance between the estimand recorded in the protocol and the estimand the published conclusions actually support; (iii) a lower frequency of causal or comparative-effectiveness claims unsupported by the available phenotype, as judged by blinded independent assessors; and (iv) acceptable feasibility. The hypothesis is refuted if projects using the documentation show no measurable difference in (i) to (iii) relative to contemporaneous or historical controls, or if independent mentor–methodologist pairs given the same protocol cannot agree on where the refusal point falls, which would show that the construct is not reproducibly identifiable. A programme designed to test and potentially refute these predictions is set out in the Discussion.

## Methods

This Hypothesis and Theory article was developed in four stages: a survey of recent oculoplastic real-world-data studies; a structured comparison of reporting and causal-inference frameworks; deductive and inductive derivation of the framework; and retrospective application of the framework to a completed study. The article is a conceptual synthesis rather than a systematic review or meta-analysis, and the basis of literature selection is stated explicitly, as recommended for non-systematic reviews (5). The work uses no patient-level data and required no ethical approval.

### Comparator studies

The studies in **Table 1** were not identified by a systematic search. They emerged from the authors’ ongoing reading of the oculoplastic and general ophthalmology literature, together with adjacent otolaryngology and head-and-neck reports, and were assembled once the recurring pattern of interest had become apparent. We did not pre-specify search terms, screening criteria, or a search date, and we did not record a study flow diagram. Included studies address an oculoplastic diagnosis or procedure using claims, administrative, registry, or federated electronic-health-record data, and were published between 2019 and 2026. In choosing among the studies available to us we sought variation in data source, and **Table 1** draws on a federated electronic-health-record network, national claims and Medicare data, a population cancer registry, and an audited surgical-quality registry.

**Table 1:** Recent oculoplastic database studies and the methodological lesson they teach.

Example	What the database can study	What was not available or not adjudicated as reported	Question for the pre-analysis review
Cannabis and thyroid eye disease in TriNetX (13)	Coded cannabis exposure and TED manifestations in autoimmune hyperthyroidism.	Smoking intensity, thyroid control, Clinical Activity Score, proptosis, diplopia grade, optic neuropathy, imaging.	Disease association, severity proxy, or residual confounding by smoking and utilization?
Endoscopic vs external DCR in TriNetX (14)	Temporal trends, approach selection, coded revision rates.	Obstruction site, laterality, ostium anatomy, canalicular disease, stent use, symptom severity, functional epiphora.	Does repeat surgery represent anatomical failure, staged care, or revision threshold?
Ptosis reoperation after intraocular surgery in TriNetX (15)	Coded ptosis repair followed by later coded reoperation.	MRD1, levator function, Hering effect, laterality, cataract-related eyelid change, cosmetic vs functional indication.	Is a second code true recurrence, contralateral surgery, refinement, or a utilization endpoint?
Blepharoptosis and falls in TriNetX (16)	Association between coded blepharoptosis and coded falls or fractures.	Visual-field obstruction, chin-up posture, frailty phenotype, neurologic disease, correction status.	Is ptosis the exposure, a marker of frailty, or a marker of health-care contact?
GLP-1 receptor agonists and oculofacial procedures in TriNetX (17)	Periorbital diagnoses and procedure utilization after systemic drug exposure.	Weight-loss trajectory, facial volume change, aesthetic demand, insurance status, cosmetic procedure capture.	Does the outcome reflect tissue change, access, or patient preference?
Orbital fracture antibiotics in TriNetX (18)	Antibiotic exposure and subsequent coded orbital or periorbital cellulitis.	Fracture severity, sinus involvement, wound contamination, operative complexity, clinician concern.	Can confounding by indication be reduced enough to support a practice conclusion?
CMS/Medicare orbital decompression studies (19) (20),	Population-level volume, approach, spending, specialty distribution after teprotumumab approval.	Disease activity, compressive optic neuropathy, diplopia, cosmetic indication, prior therapy, individual response.	Is reduced volume a change in severity, pathway, referral, or coding?
Medicare DCR trend study (21)	Regional and temporal trends in external vs endoscopic DCR.	Anatomical success, symptom relief, quality of life, endoscopic findings, revision indication.	Is the administrative trend clinically meaningful without phenotype or outcome adjudication?
Eyelid sebaceous gland carcinoma in SEER (22)	National incidence, stage at diagnosis, demographics, relative survival over two decades.	Margin status, Muir–Torre association, pagetoid spread, Mohs/map-biopsy clearance, reconstruction, local recurrence.	Does registry survival reflect tumor biology or unrecorded completeness of excision and reconstruction?
Orbital floor fracture repair in NSQIP (23)	Validated 30-day systemic outcomes (length of stay, infection, sepsis) by emergent vs non-emergent status.	Entrapment, motility, diplopia, enophthalmos, defect size, functional ophthalmic result.	Is a 30-day systemic-safety endpoint the same construct as a successful orbital repair?

CMS, Centers for Medicare & Medicaid Services; DCR, dacryocystorhinostomy; GLP-1, glucagon-like peptide-1; MRD1, margin-reflex distance 1; NSQIP, National Surgical Quality Improvement Program; SEER, Surveillance, Epidemiology, and End Results; TED, thyroid eye disease.

The ten studies are illustrative cases identified non-systematically, as described in the Methods; they are not a systematic or representative sample, and no prevalence claim should be drawn from them. The row for orbital decompression combines two related national analyses, so that ten studies correspond to eleven references. Entries in the third column denote variables that were not available, not analysed, or not adjudicated as reported in the published study and in publicly available documentation of the data source; the classification categories are defined in the Methods. They do not imply that the investigators could have obtained these variables or should have analysed them, and several were not required for the studies’ own stated aims. The NSQIP and SEER entries are retained as counterexamples in which the reported constructs are adequately measured.

### Classification of variables reported as unavailable

For each included study we read the published report in full and, where we needed it, consulted publicly available documentation of the underlying data source, such as variable lists and registry manuals. Each variable listed in **Table 1** was then classified as absent from the data source; present in the source but not analysed or not reported; present but of uncertain measurement validity; or not required for the study’s own stated aim. We did not have access to the original protocols, coding algorithms, analytic code, linked datasets, or supplementary materials of the included studies. **Table 1** therefore reports variables that were not available or not adjudicated *as reported*.

### Frameworks

We examined established reporting and causal-inference frameworks applicable to routinely collected health data: STROBE, RECORD, CODE-EHR, IDEAL, the ICH E9(R1) estimand framework, and target trial emulation, together with general fitness-for-purpose and construct-validity assessment. For each we extracted what the framework explicitly requires, what it leaves to investigator discretion, and whether it specifies an owner, a timing, a storage location, or an audit mechanism for the decision that a dataset cannot support an intended inference. The extraction is reported in **Table 2**.

**Table 2:** What existing frameworks require, what remains unspecified, and what the refusal point adds.

Framework	What it already requires	What it leaves unspecified	What the refusal point adds
STROBE (6)	Transparent reporting of design, setting, participants, variables, sources of bias, and limitations in observational studies.	Whether the data can support the intended inference; no owner, timing, or storage is specified for that judgment, and limitations are written after the analysis.	Moves the limitation statement to before data extraction and attaches dated, named signatories to it.
RECORD (7)	Reporting of code lists, data linkage, validation, cleaning, and population selection for routinely collected data.	Whether a correctly reported code is an adequate surrogate for the clinical construct at issue in this question.	Requires the construct–surrogate gap to be stated for this question and this dataset, in clinical terms.
CODE-EHR (8)	Minimum standards for data quality, provenance, linkage, and governance in structured electronic health-record research.	The subspecialty-specific clinical content of the gap, and the decision not to proceed.	Supplies the oculoplastic content of the gap and records the decision not to proceed as a deliverable.
IDEAL (9)	Stage-appropriate evaluation of surgical innovation, with explicit attention to operator, learning curve, and indication.	What to do when a dataset cannot represent indication, technique, or surgeon threshold at all.	Names in writing the specific surgical question the source cannot adjudicate.
ICH E9(R1) estimand (2)	Precise specification of population, treatment, endpoint, intercurrent events, and population-level summary.	What follows when the specified estimand is not estimable in the available data; who decides, and where the decision is recorded.	Documents the non-estimable adjacent estimand and the design change it forces.
Target trial emulation (3) (4),	Explicit specification of the hypothetical trial: eligibility, treatment strategies, assignment, time zero, follow-up, and outcome.	Whether the data contain the clinical variables the emulated trial requires, and disclosure when they do not.	Records the specific clinical variables whose absence prevents the emulation, before extraction.
Fitness-for-purpose and construct-validity assessment (general practice)	Data are assessed for relevance and reliability for the intended use.	Usually informal, undated, unattributed, not stored with the protocol, and not auditable against the published paper.	Converts an informal assessment into a dated, co-signed, filed, auditable artifact.

Who documents: the clinical expert, the trainee or lead analyst, and a methodologist or data scientist, as co-signatories. When: before data extraction. Where: filed with the study protocol and, where institutionally supported, preregistered. How adherence is evaluated: concordance between the filed map and the estimand the published conclusions actually support, checkable by supervisors, reviewers, and editors.

### Framework derivation

The framework was developed by the three authors: an oculoplastic surgeon responsible for research supervision (ZG) and two clinician-investigators with training in epidemiology and public health and prior experience of federated electronic-health-record analysis (IN, TS). The layers and the components of the map were derived in two directions: deductively, from the requirements of the frameworks in **Table 2**, and inductively, from the recurring failure modes observed when those requirements were applied to the studies in **Table 1**. The draft was revised over successive rounds of discussion among the authors, in which a disputed component was retained only if we could identify a design decision it would have changed. The map was then applied retrospectively to a completed study by the authors as an internal pilot. We did not conduct a formal consensus process, expert interview study, or Delphi exercise; the framework has not been reviewed by independent external experts and has not been applied prospectively.

## Results

### What existing frameworks require, and what remains unspecified

Reporting frameworks have improved observational research: STROBE clarified expectations for observational studies, RECORD extended them to routinely collected health data, and CODE-EHR proposed minimum standards for structured electronic records (6–8) The IDEAL recommendations address a further problem specific to surgical research: procedures are operator-dependent, evolve over time, and are shaped by learning curves, indication, technique, setting, and postoperative thresholds (9). These frameworks can require transparent codes, eligibility criteria, and analytic choices, but they cannot determine whether, for example, a code for ptosis repair represented functional obstruction, aesthetic asymmetry, myogenic ptosis, aponeurotic dehiscence, Hering effect, revision, or staged care. The IRIS Registry illustrates the value of specialty-specific data at scale, yet even ophthalmic registries depend on what is structured, captured, and validated (10). Validation studies show that code agreement varies by disease and is weaker for broader or less severe categories (11), and a narrative review of TriNetX in ophthalmology cautioned that coding accuracy, completeness, matching, and bias require careful design (12). For oculoplastics, the decisive variable is often not the billing code but laterality, measurement, image, examination finding, operative nuance, surgeon threshold, or patient-reported state, all closer to phenotype and judgment than to code.

The availability of these layers varies between data sources and between constructs. Validated code algorithms can achieve high positive predictive value for well-demarcated conditions; structured fields can carry laterality and measurement; natural-language processing of operative notes and pathology reports can recover phenotype; and audited registries can supply adjudicated outcomes. Conversely, a recorded clinical measurement may be incomplete, inconsistently obtained, or unreliable. Whether a source can carry a given construct therefore depends on its provenance, completeness, measurement validity, temporal accuracy, and external validation for that construct.

**Table 2** sets out this comparison. Each of these frameworks requires that the question be well posed and the data adequate to it. None names the person accountable for the judgment that the data are not adequate, fixes the point in the workflow at which that judgment is recorded, or makes it auditable against the paper eventually published.

### Where the boundary falls in recent oculoplastic real-world data studies

Applying this lens to ten recently published oculoplastic database studies (**Table 1**) shows a consistent pattern. A TriNetX study of cannabis use and thyroid eye disease can test a coded association but remains constrained by smoking behavior, thyroid control, activity, and severity (13). A comparison of endoscopic and external dacryocystorhinostomy (DCR) can evaluate trends and coded revision rates, but a revision code does not equal anatomical failure, persistent epiphora, canalicular disease, or dissatisfaction (14). A study of reoperation after intraocular surgery in eyelids previously repaired for ptosis, our own work, can quantify coded reoperation but cannot separate true recurrence from contralateral Hering effect, planned sequencing, cosmetic refinement, or surgeon threshold (15); under the present framework, it is defensible only as a hypothesis-generating description of a coded endpoint. A study of blepharoptosis and falls can test a population-level association without revealing visual-axis obstruction, posture, field loss, or frailty (16). Systemic-drug and trauma studies repeat the lesson: analyses of glucagon-like peptide-1 receptor agonists and oculofacial procedures detect utilization signals but cannot distinguish biologic tissue change from weight loss, aesthetic demand, or coding of cosmetic procedures (17), and an orbital-fracture antibiotic study is intrinsically exposed to confounding by indication (18). Medicare and CMS analyses of orbital decompression around teprotumumab approval demonstrate changing volume, approach, and spending but not inflammation, compressive optic neuropathy, or operative intent (19, 20), and Medicare DCR analyses show practice patterns but not ostium anatomy, lacrimal pump function, or quality of life (21). Disease and surgical-quality registries show the same boundary from different ecosystems: a SEER analysis of eyelid sebaceous gland carcinoma describes incidence and survival but not margin status, pagetoid spread, clearance, or reconstructive burden (22), and an NSQIP analysis of orbital floor fracture repair captures validated 30-day systemic outcomes but neither the orbital phenotype nor the functional ophthalmic result (23).

Two of these studies are retained deliberately as counterexamples, because the boundary in each falls late rather than early. The NSQIP analysis (23) reports outcomes abstracted by trained reviewers against standardized definitions and subject to inter-rater audit, so its 30-day systemic endpoints are adjudicated rather than inferred from codes; the limitation concerns which construct was chosen, not how well it was measured. The SEER analysis (22) likewise reports incidence, stage distribution, and relative survival, which are constructs a population cancer registry is designed to measure and measures well; the refusal point applies only if the intended conclusion moves to completeness of excision or local recurrence. Adequately captured phenotype is therefore attainable within routinely collected data, and the framework is intended to identify where the boundary falls in a given study rather than to assume a fixed position for it.

### Recurring ambiguous constructs

Across these examples, reoperation, revision, and diagnosis codes recur as structurally ambiguous outcomes. Reoperation after ptosis repair may represent failure, planned contralateral surgery, under- or overcorrection, contour dissatisfaction, exposure risk, or preference; revision after DCR may reflect anatomical closure, functional epiphora, canalicular pathology, stent issues, or surgeon threshold; and a diagnosis of thyroid eye disease may span mild inactive disease, active inflammation, compressive optic neuropathy, diplopia-dominant disease, or fibrotic sequelae. Some questions suit routinely collected data, as demonstrated above: procedure trends, medication-safety signals, rare associations, utilization, access disparities, and long-term coded revision. Others require phenotype-rich data: laterality, margin-reflex distance 1 (MRD1), levator function, proptosis in millimeters, Clinical Activity Score, operative detail, imaging, pathology, and patient-reported outcomes. A third group – subtle aesthetic success, true anatomical recurrence, technical quality, and satisfaction – should not be answered by codes alone in the absence of validation, linkage, or adjudication, as the BOPSS National Ptosis Survey and studies of functional indication and patient-reported benefit demonstrate (24–28).

Nothing in this classification implies that claims or registry data are intrinsically unsuitable for comparative-effectiveness research. Whether a source can support a comparative claim depends on the specific estimand, the validation status of the codes used, the availability of linkage, the design, and the sensitivity analyses performed; the same source may be adequate for one estimand and inadequate for another. The classification concerns the fit between a question and a dataset rather than the relative quality of data sources.

## Discussion

### Mentorship as metadata: metaphor, artifact, and ownership

In data science, metadata describes data and makes it interpretable; without it, a field of values has no defined meaning, provenance, or permissible use. In oculoplastic education, mentorship performs an analogous function, teaching the context around the code: what the diagnosis included, what the operation intended, what the outcome meant, and what the dataset omitted.

The analogy has limits. Formal metadata are standardized, persistent, structured, reproducible, and often machine-readable; formal data provenance and research-governance documentation are institutional records with defined custodianship and retention. Mentorship is none of these. It is interpersonal, variable, and shaped by authority, experience, and local culture. We therefore distinguish the artifact from the relationship that produces it and from the analogy that motivates it. The artifact proposed here is documented clinical interpretive annotation: a written, dated, co-signed record of the clinical limits of a dataset for a stated question, filed with the protocol. It is produced through clinical supervision, and the metadata analogy describes why such a record is needed. The deliverable is the annotation, and the framework does not depend on the analogy.

This role is narrower than mentorship’s usual remit, which spans skill, identity, research development, and well-being (29). We add a specific research-education task: the mentor teaches methodological restraint, reviewing the phenotype map, challenging the code list, identifying missing variables, anticipating bias direction, requiring validation, and defining the refusal point before results exist (**Figure 1**).

**Figure 1:**
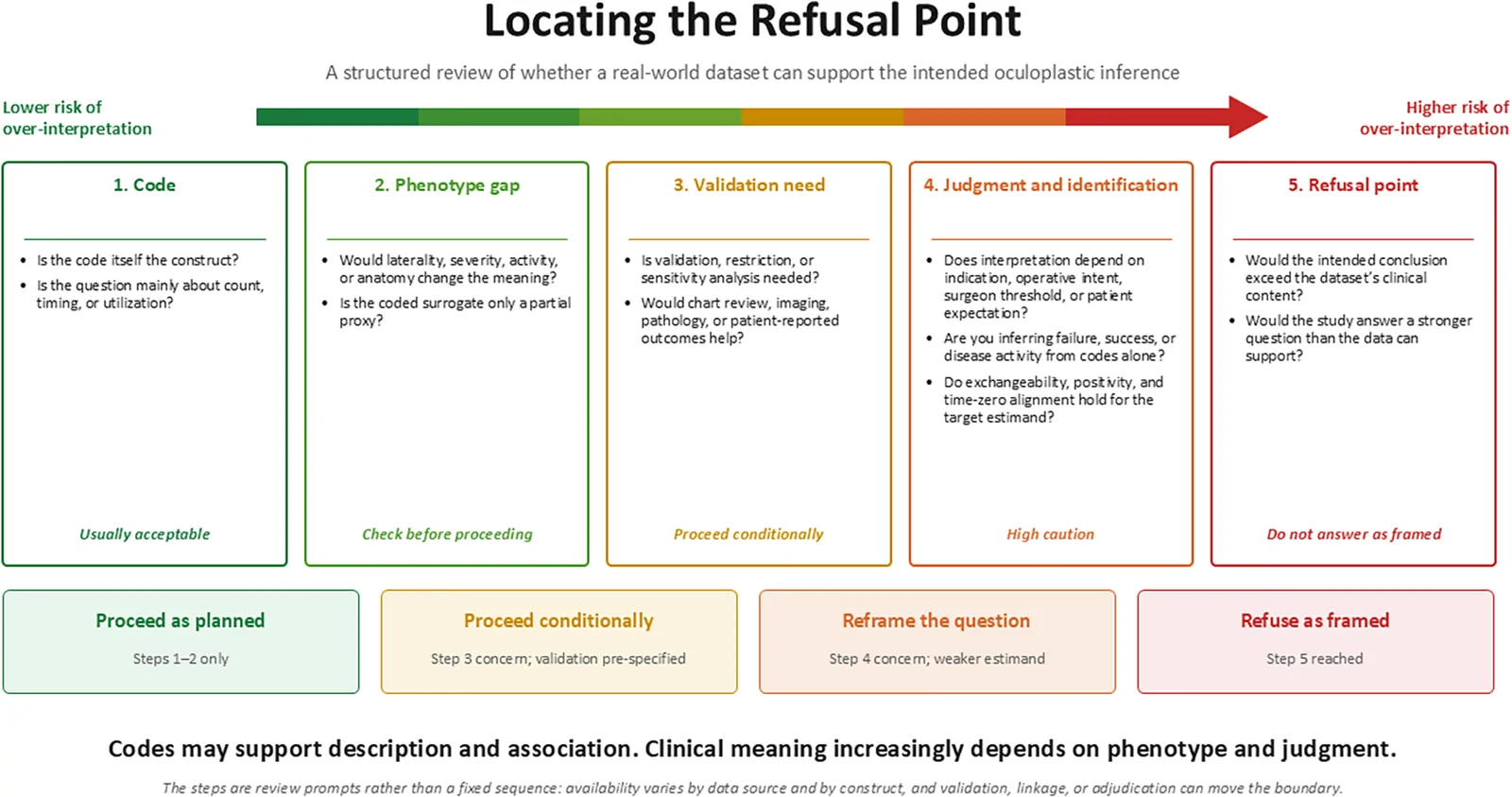
Locating the refusal point in oculoplastic real-world data research. This decision aid illustrates how the permissible strength of inference changes as a question moves from code-level description to phenotype-dependent and judgment-dependent interpretation. Codes may support description, utilization, timing, and coded associations; claims about clinical meaning require phenotype-rich or adjudicated data. The refusal point is reached when the intended conclusion exceeds the dataset’s clinical content, after which the question should be validated, reframed, or not answered as framed. The four decision grades shown (proceed as planned; proceed conditionally; reframe the question; refuse as framed) correspond to the minimum criteria specified in the Discussion, and the grade assigned is recorded in the pre-analysis map (**Table 4**). The steps are review prompts rather than a fixed sequence, and validation, linkage, or adjudication can move the boundary.

The educational literature supports both the value and the hazard of locating this task with a senior clinician. Mentoring is consistently associated with research productivity and career development, although that evidence base is dominated by cross-sectional self-report and remains methodologically weak (30). At the same time, a steep authority gradient is a recognized contributor to error precisely because it suppresses challenge from the less senior party (31), and the cognitive dispositions most relevant here (anchoring on the available code, confirming the hypothesis the trainee arrived with, and premature closure on an attractive comparative claim) are those for which externalized, structured checks outperform individual vigilance (32). A written refusal point is therefore best understood as a cognitive forcing function applied to study design: it requires the team to state the unanswerable version of the question before any result is available. Competency in this task is assessable, since a trainee can be asked to produce a defensible map for a protocol they did not write, and we regard that as a more tractable educational endpoint than mentorship quality in general.

#### Ownership and disagreement

The refusal point should not be owned by the oculoplastic mentor alone. A senior clinician may have no formal training in causal inference, database architecture, measurement error, or statistical modelling, and exclusive senior ownership risks exactly the authority gradient described above. The refusal point should be jointly reviewed and co-signed by at least three parties: the clinical expert, accountable for the clinical content of the boundary; the trainee or lead analyst, accountable for the operational definitions; and a methodologist or data scientist, accountable for the identification conditions. Where they disagree, the disagreement is recorded rather than settled by seniority. Each signatory may enter a dated dissenting statement in the map; the more restrictive position governs the analysis by default; and unresolved disagreement is escalated to a departmental research committee or an independent methodologist. A refusal point may be revised after signature, but only by dated amendment stating what changed, why, and at whose request, so that any relaxation of the boundary is visible to reviewers rather than silent.

Two worked examples show the discipline changing a conclusion rather than merely qualifying it. First, a trainee proposes to use a claims network to show that endoscopic DCR is “superior” to external DCR by a lower coded revision rate. The phenotype map forces three admissions: revision is a utilization code, not an adjudicated failure; external and endoscopic DCR may be embedded in different specialty pathways; and obstruction site and symptom severity are unmeasured, producing confounding by indication of unknown direction. The refusal point is reached for the word “superior,” and the study is downgraded to a descriptive, hypothesis-generating account of trends. Second, a fellow asks whether teprotumumab approval changed orbital decompression practice. The causal question (did the drug reduce the need for decompression in active disease)? is unanswerable without Clinical Activity Score or optic-neuropathy data, so the refusal point is written against it; but naming it clarifies a legitimate, well-specified utilization estimand: the population-level rate, approach, and cost of coded decompression before and after a fixed approval date, converting a vulnerable comparative-effectiveness study into a defensible interrupted-time-series description. The discipline is therefore not only subtractive; it relocates a project onto the firmest construct the data can carry.

### A three-layer model and a pre-analysis phenotype map

Every oculoplastic database project can be taught through three layers (**Table 3**): the code (diagnosis, procedure, medication, test, encounter, date); the phenotype (severity, laterality, anatomy, activity, function, imaging, histology, patient-reported symptoms); and judgment (indication, expectation, operative plan, surgeon threshold, revision tolerance, interpretation of success or failure). In many routinely collected sources availability decreases across these layers, although the layers classify what a question requires rather than ranking what a source contains, and availability varies by source and by construct as described above. **Table 3** states what typically strengthens each layer. If the question concerns utilization, the code may be the construct; if it concerns severity, the code is a proxy; if it concerns aesthetic success, operative quality, or the meaning of revision, the dataset must contain images, operative notes, structured measurements, patient-reported outcomes, or validation. This is not anti-database thinking but better database thinking, protecting against both dismissing large datasets because they are imperfect and accepting them because they are large.

**Table 3:** Three-layer model for oculoplastic database research.

Layer	What is usually available	What documented clinical review adds	What typically strengthens this layer
Code	Diagnosis, procedure, medication, laboratory, imaging order, encounter, date.	Tests whether the code is a valid surrogate for the clinical construct.	Published validation of the code algorithm in a comparable population; reported positive predictive value; local validation against a reference standard.
Phenotype	Severity, laterality, anatomy, activity, function, image, pathology, patient-reported symptoms, variably captured.	Identifies missing phenotype and decides whether validation, restriction, or phenotype-rich data are required.	Structured measurement fields; imaging and pathology linkage; natural-language processing of operative notes; validated patient-reported outcome instruments; chart or photograph review of a random subsample.
Judgment	Indication, expectation, operative plan, surgeon threshold, revision tolerance, meaning of success or failure, rarely captured.	Defines the interpretation boundary and the refusal point, co-signed by clinical, analytic, and methodological reviewers.	Prospective capture of indication and operative intent; adjudication committees; surgeon-completed structured fields; linked survey or qualitative data. Where none is available, the boundary is the refusal point.

Each project should begin with a clinical map before a statistical query (**Table 4**), defining the target construct, the coded surrogate, the measurement validity of that surrogate, the expected missing variables, the likely bias direction, the identification conditions required by the target estimand, the outcome class, the minimum validation required, and the refusal point. Naming the refusal point in advance converts a vague awareness of limitations into a concrete design decision, and the co-signatories should review the map before the first result is generated. The map prevents outcome drift (a revision code is a utilization outcome, not automatically a failure outcome; steroid exposure is a treatment proxy, not a disease-activity measure) and links clinical judgment to analysis, because propensity-score matching can balance recorded variables but not variables that were never measured.

**Table 4:** Pre-analysis phenotype map for oculoplastic real-world data studies.

Step	Required decision before analysis	Reason
Target construct	Define the clinical state, operation, exposure, or outcome the study is actually trying to measure (the estimand).	Prevents substituting a convenient code for the intended clinical question.
Coded surrogate	List diagnosis, procedure, medication, laboratory, imaging, encounter, and exclusion codes; specify index date and laterality availability.	Makes the operational definition reproducible and reviewable.
Measurement validity	State the validation status of each code or algorithm (published sensitivity or positive predictive value in a comparable population, local validation, or none) and the temporal accuracy of the index date.	Distinguishes a measured construct from an assumed one.
Phenotype gap	Identify decisive variables that are absent or inconsistently captured (MRD1, levator function, proptosis, activity, histology, indication, patient-reported state).	Identifies where misclassification is likely.
Bias direction	State whether missing severity, indication, follow-up, threshold, or utilization would make the association larger, smaller, or indeterminate. Where indeterminate, specify the negative control, quantitative bias analysis, sensitivity measure, or validation subsample that will be used.	Turns limitation reporting into study design without forcing false precision about direction.
Identification conditions	For the target estimand, state whether exchangeability, positivity, consistency, and alignment of eligibility, treatment assignment, and time zero are plausible, and how each will be assessed.	Separates whether the data measure the construct from whether the design can identify the effect.
Outcome class	Classify each outcome as anatomical, functional, psychosocial, utilization-based, safety-related, or economic.	Prevents interpreting utilization as failure, satisfaction, or disease activity.
Analytic implication	Define unit of analysis, time zero, lag, censoring, competing events, clustering, and measured confounders before querying.	Links clinical validity to statistical validity.
Validation need	Decide whether chart, photograph, operative-note, pathology, image review, or patient-reported outcome data are required.	Separates hypothesis generation from outcome adjudication.
Refusal point	Write the version of the question that should not be answered by this dataset, record the decision grade (proceed, proceed conditionally, reframe, or refuse as framed), and obtain dated signatures from the clinical expert, the trainee or lead analyst, and the methodologist, with any dissent recorded.	Makes non-analysis a valid, jointly owned and auditable methodological decision.

MRD1, margin-reflex distance 1.

#### Two distinct tests

Phenotype misclassification, unmeasured confounding, selection bias, confounding by indication, and causal overinterpretation are related but methodologically distinct. They fail for different reasons and have different remedies, and the map separates them into two tests. The first is a *measurement* test: does the source measure the construct named in the estimand, with acceptable sensitivity, specificity, and temporal accuracy, and is the missing-data mechanism understood? Failure here is addressed by validation, restriction, linkage, adjudication, or by redefining the construct. The second is an *identification* test: for the target estimand, are exchangeability, positivity, consistency, and alignment of eligibility, assignment, and time zero plausible, and how will each be assessed? Failure here is not repaired by better measurement; it is addressed by design (active comparator, new-user restriction, negative controls, quantitative bias analysis) or by retreating from a causal to a descriptive estimand. A dataset may pass one test and fail the other. Accurate measurement with unverifiable exchangeability supports description but not causal contrast; adequate identification with a poorly measured outcome supports a causal statement about a surrogate rather than about the clinical construct. The refusal point is reached when either test fails for the intended conclusion and no available remedy closes the gap.

#### Operationalizing the threshold

The decision is graded rather than binary, and **Figure 1** sets out the grades; we specify here the minimum criteria for each. A project *proceeds as planned* when the coded surrogate either is the construct or has published validation in a comparable population, and the identification conditions for the stated estimand are plausible and testable. A project *proceeds conditionally* when a decisive phenotype variable is missing or of unknown validity but a defined procedure could establish whether the gap is material; the procedure (chart or photograph review of a random subsample, code validation against a reference standard, negative-control analysis, or quantitative bias analysis) must be pre-specified in the protocol rather than promised in the discussion. A project is *reframed* to a weaker but supportable estimand when the measurement test passes and the identification test fails, or when a utilization construct has been informally treated as a clinical one. The *refusal point* is reached, and the question is not answered as framed, when the intended conclusion depends on a variable that is absent from the source and cannot be recovered by validation, linkage, or adjudication within the study. Because the grade assigned to any individual project depends on its estimand, these criteria do not remove judgment, but they make it explicit, dated, attributable, and reviewable. The completed map should be filed with the protocol and, where the institution supports it, preregistered. Retrospective change requires a dated amendment recording what changed, why, and which signatory requested it, and the final manuscript should be checkable against the filed map.

#### When the direction of bias is unknown

For many unmeasured confounders the direction and magnitude of bias are genuinely uncertain, and requiring a directional answer would invite false precision. The map therefore permits three answers (larger, smaller, or indeterminate) and requires that “indeterminate” be accompanied by a plan: a negative-control exposure or outcome (33), a quantitative or probabilistic bias analysis, an E-value or equivalent sensitivity measure for the reported estimate (34), or a validation subsample. An indeterminate direction recorded with a sensitivity plan avoids assigning a direction the data cannot support.

#### A worked example

To show that the map is usable rather than merely describable, **Table 5** presents it fully completed, applied retrospectively to our own published study of reoperation for ptosis after intraocular surgery (15). We used our own work for this purpose deliberately. It permits a fully candid appraisal without attributing unstated intentions to other investigators, and it applies the framework’s most restrictive criteria to the authors before applying them to anyone else. The same classification criteria were applied to all ten studies in **Table 1**, but only for our own study do we have access to the design reasoning behind the published methods, which is why the completed map is presented for that study alone. The exercise downgrades the defensible claim of that paper from recurrence to coded reoperation.

**Table 5:** The pre-analysis phenotype map completed retrospectively for a published study by the authors (15).

Step	Completed entry
Target construct	Intended: whether intraocular surgery causes true recurrence of ptosis in eyelids previously repaired. Available: a second coded ptosis-repair procedure in patients who underwent a first coded intraocular surgery in the interval following an index coded ptosis repair, compared with patients with no coded intraocular surgery in that interval.
Coded surrogate	Procedure and diagnosis codes for ptosis repair and for intraocular surgery within a federated electronic-health-record network; index date defined as the first coded ptosis repair; exposure defined by a coded intraocular surgery within a fixed window after the index repair; laterality not reliably available.
Measurement validity	No published validation of the ptosis-repair code algorithm against chart-confirmed recurrence in this population. Temporal accuracy of the index date acceptable. Laterality unverifiable, so ipsilateral and contralateral procedures cannot be distinguished.
Phenotype gap	MRD1, levator function, pre- and postoperative photographs, contralateral Hering effect, planned staged or contralateral surgery, cosmetic versus functional indication, individual surgeon threshold for revision.
Bias direction	Indeterminate. Contralateral and staged procedures inflate the coded reoperation count; revisions performed outside the network deflate it, and the two act in opposite directions with unknown relative magnitude. Plan: negative-control outcome (an unrelated eyelid procedure), sensitivity analysis excluding reoperations within 90 days, and an E-value for the reported estimate.
Identification conditions	Positivity plausible. Exchangeability not verifiable, because indication for the index repair and surgeon revision threshold are unmeasured. Time zero definable. A causal contrast is therefore not identified.
Outcome class	Utilization. Not anatomical, not functional, not psychosocial.
Analytic implication	Patient-level unit of analysis; time zero at index repair; censoring at last network encounter; site-level clustering unmodelled.
Validation need	Chart and photograph review of a random subsample would be required to convert coded reoperation into adjudicated recurrence. Not available in this data source.
Refusal point	Do not conclude that intraocular surgery causes recurrent ptosis, and do not report the coded reoperation rate as a recurrence rate. Decision grade: reframe. Defensible claim: a hypothesis-generating description of the rate and timing of coded reoperation after coded ptosis repair in this population.
Signatories	Clinical expert (ZG), lead analyst (IN), methodologist (TS). No dissent recorded. Completed retrospectively as an internal pilot.

Completed by the authors for their own published study as an internal pilot of the map. It is a retrospective self-assessment and not an independent evaluation. The exercise downgrades the defensible claim of the published paper from recurrence to coded reoperation. MRD1, margin-reflex distance 1.

### Practical implications for training

This approach requires not a new curriculum but a new habit, and we present it as a proposal, not a tested intervention. Each project should include a short pre-analysis conference at which the trainee presents the target phenotype, code strategy, missing variables, anticipated biases, outcome class, validation plan, and refusal point; the oculoplastic mentor tests the clinical logic, and the methodologist tests the analytic logic, with **Table 4** serving as a single-page template that all three parties sign. Departments should encourage paired mentorship because a methodologist understands confounding and censoring, while an oculoplastic surgeon understands why a second operation is ambiguous, why laterality matters, why MRD1 is not interchangeable with a diagnosis code, and why satisfaction is not technical success. Trainees should also be taught that refusing certain questions is maturity, not failure: the most honest conclusion may be that a database can generate a hypothesis but cannot adjudicate the clinical outcome. Trainees should be told explicitly that recording dissent from a supervisor’s judgment in the map is a normal use of the document rather than a challenge to supervision.

### A prospective validation programme

The hypothesis stated in the Introduction is testable, and we propose the following programme rather than leaving validation as an aspiration. *Design:* a prospective, two-centre implementation study of consecutive oculoplastic real-world-data projects over 24 months, with a historical control set of projects completed in the same departments during the preceding 24 months, and blinded outcome assessment. *Primary outcome:* concordance between the estimand recorded in the pre-analysis map and the estimand actually supported by the conclusions of the resulting manuscript, rated independently by two assessors blinded to allocation. *Secondary outcomes:* inter-rater agreement (Cohen’s κ) on refusal-point placement between independent mentor–methodologist pairs given the same protocol, as a direct test of whether the construct is reproducibly identifiable; the proportion of projects in which the research question was reframed before data extraction; the frequency of causal or comparative language unsupported by the available phenotype, scored by blinded assessors against a pre-specified rubric; completeness of reporting against RECORD; trainee performance on a short pre- and post-implementation assessment of data-fitness reasoning; and feasibility, measured as completion time, number of amendments, and participant-reported burden. *Pre-specified refutation criteria:* the framework fails if κ for refusal-point placement is below 0.4, if the primary outcome does not differ from control, or if median completion time exceeds 60 minutes per project, which would make routine use implausible. We present the framework as a hypothesis to be tested rather than as a validated instrument, and regard this programme as the next step.

### Limitations

This framework is proposed, not validated. Its central act overlaps conceptually with the estimand and target-trial frameworks it complements; our claim rests on direction, ownership, and deliverable status rather than on a new statistical construct, and a department already writing rigorous estimands will find the refusal point a small, natural addition. It was derived by three authors at a single institution without formal consensus methodology, expert interviews, or external review, and it has not been applied prospectively; the internal pilot in **Table 5** is retrospective and was performed by the authors on their own work, which cannot substitute for independent assessment. Mentorship is variable and institution-specific, and mentors may transmit local assumptions unless challenged by transparent reporting, validation, statistical review, and collaboration. The **Table 1** studies were selected to illustrate the boundary and are not exhaustive. Their selection was non-systematic and was made by clinicians who had already formed a view, so the set remains susceptible to confirmation bias despite the retention of counterexamples. The studies are illustrative cases rather than evidence for the framework, and no conclusion about how frequently the problem occurs in the oculoplastic literature should be drawn from them; a claim of that kind would require a systematic review, which this article does not attempt. Our appraisal of individual studies rests on published reports and publicly available source documentation rather than on original protocols, coding algorithms, or analytic code, and may therefore misclassify variables that were available but unreported, or unnecessary for those studies’ own aims. The model should complement, not replace, RECORD, CODE-EHR, IDEAL, the estimand and target-trial frameworks, chart and photograph review, natural-language extraction, or patient-reported outcome measurement. As structured templates, image-linked registries, surgical video, and multimodal phenotyping make currently uncoded variables measurable, the refusal point will move rather than disappear; the central principle is unchanged: the research question must match the data source.

## Conclusion

Real-world data will continue to shape oculoplastic research. Their value (scale, access, efficiency, long follow-up, rare-event detection) is substantial, but they often miss the variables that make oculoplastic surgery oculoplastic. The future surgeon-scientist should be trained not only to operate and to analyze data, but to recognize the boundary between code and clinical reality and to write that boundary down before analysis begins. We have also specified how this proposal could be tested and refuted. In oculoplastics, mentorship is metadata: the documented clinical annotation that allows data to become evidence.

## Data Availability

The original contributions presented in the study are included in the article/supplementary material. Further inquiries can be directed to the corresponding authors.
